# Antibacterial Peptide HHC-36 Sustained-Release Coating Promotes Antibacterial Property of Percutaneous Implant

**DOI:** 10.3389/fbioe.2021.735889

**Published:** 2021-09-27

**Authors:** Qiang Miao, Jin-Long Sun, Fei Huang, Jing Wang, Pei Wang, Ya-Fei Zheng, Feng Wang, Chu-Fan Ma

**Affiliations:** ^1^ Department of Stomatology, Sixth Medical Center of PLA General Hospital, Beijing, China; ^2^ State Key Laboratory of Military Stomatology, National Clinical Research Center for Oral Diseases, School of Stomatology, The Fourth Military Medical University, Xi’an, China; ^3^ Shaanxi Key Laboratory of Stomatology, School of Stomatology, The Fourth Military Medical University, Xi’an, China; ^4^ Air Force Medical Center, The Fourth Military Medical University, Beijing, China; ^5^ Department of Bone and Joint Surgery, Wendeng Orthopaedic Hospital of Shandong Province, Wendeng, China

**Keywords:** antibacterial peptide, HHC-36, percutaneous implant, antibacterial property, titanium dioxide nanotube (TNT)

## Abstract

Percutaneous implants are widely used in clinical practice. However, infection is the main clinical problem of percutaneous implants. Titanium dioxide nanotubes are suitable for forming coatings on complex surfaces such as implants. HHC-36, a cationic antimicrobial peptide, has been identified to have a strong broad-spectrum antibacterial effect. In the present study, we use poly D,L-lactic acid (PDLLA) and poly lactic-co-glycolic acid (PLGA) coating to build HHC-36 sustained-release system on the surface of titanium dioxide nanotubes. The titanium specimens were anodized coated with HHC-36-PDLLA/PLGA. The morphology and surface elemental distribution of the specimens were evaluated. Besides, results in the present study demonstrated that with antibacterial peptide HHC-36 sustained-release coating, titanium dioxide nanotubes maintain effective drug release for 15 days *in vitro*, and show significant antibacterial activity. The proliferation of *Staphylococcus aureus* can be effectively inhibited by PDLLA/PLGA-HHC-36 coated titanium dioxide nanotube. In addition, PDLLA-HHC-36 and PLGA-HHC-36 coating was demonstrated to be biocompatible and antibacterial *in vivo*. These findings demonstrated that HHC-36 coated titanium nanotube could improve antibacterial potential of percutaneous implants, and indicated a novel and efficient strategy in preventing bacterial infection of percutaneous implants.

## Introduction

Percutaneous devices are widely used in clinical treatment in recent years, and applications include dental implants, external fixators, electrical connection of sensors, vascular access devices, auditory prostheses, and orthopedic prostheses, among others. Although the advantages of percutaneous devices are various, considerable drawbacks still bring problems in the clinical application. It has been reported that bacterial infection, abscess formation, avulsion, and extrusion are among the most common complications in percutaneous devices (Tillander et al., 2010). Bacterial infection of percutaneous implants leads to marsupialization, per-migration, and avulsion, which has been demonstrated to be the most common reason for the ultimate failure of percutaneous implants (von Recum, 1984; Xu et al., 2021). To prevent percutaneous implants from bacterial infection, proper surgical procedures, and systemic antimicrobial prophylaxis have been applied as standard in clinics over recent decades. However, for maxillofacial implants, percutaneous implants still have far higher infection rate and failure rate than oral implants, with reported infection rates ranging from 5 to 30% in bone-anchored prostheses (Tillander et al., 2010; Farrell et al., 2014a) to >50% for external fixators (Green 1983; Mahan et al., 1991). The reasons for the high infection rate include the skin exposure of the implants to the bacterial environment, and the skin accessories such as sebaceous glands, sweat glands, and sweat pores are conducive to the adhesion of bacteria.

Cationic antimicrobial peptides are produced by virtually all species of life for direct antibiotic activity or for their roles in the innate immune response (Mookherjee and Hancock 2007). These peptides are a promising source for new antibiotics, which could also be used synergistically with conventional antibiotics against multi-drug-resistant microbial infections. HHC-36 is a synthetic monomeric cationic antimicrobial peptide, which shows a broad-spectrum activity against multidrug-resistant strains of both Gram-positive and -negative bacteria and minimal toxicity against mammalian erythrocytes (Nichols et al., 2013; Lakshmaiah Narayana & Chen 2015). Though clinical application of HHC-36 is promising, effects of HHC-36 in preventing bacterial infection of dental percutaneous implants have not been previously investigated yet.

To prevent infection, recent studies have been concentrating on the development of a dynamic interface between the skin tissue and the percutaneous device. Applying specific nano-conformations on the surface (Gallo et al., 2003; Leaper 2006; Olerud, 2008; Oyane et al., 2011; Ferreira and Marais 2012; Farrell et al., 2014b) has been demonstrated to be an effective strategy. It has been reported that the structure of titanium dioxide nanotube has antibacterial properties (Su et al., 2018). In the present study, poly D,L-lactic acid (PDLLA) and poly lactic-co-glycolic acid (PLGA) coating were employed to build HHC-36 sustained-release system. We showed that percutaneous titanium nanotube implants with HHC-36 coating present an optimal antibacterial property. Thus, the results of the present study indicated a novel and efficient strategy in preventing bacterial infection of percutaneous implants.

## Materials and Methods

A flow chart depicting the sequence of experiments conducted in the present study is included in the Supplementary Information (Supplementary Figure S1).

### Titanium Nanotube Specimen Preparation

The titanium specimens were cut into round sheets with a diameter of 10 mm and a thickness of 1 mm then polished by waterproof abrasive paper of 400 meshes, 600 meshes, 800 meshes, 1,000 meshes, 1,200 meshes, and 1,500 meshes respectively. The specimens were vibrated in acetone, ethanol, and deionized water for 15 min respectively. Electrolyte for anodizing using 400 ml deionized water and 5 ml HF were mixed in magnetic stirrer. Specimens were placed at the anode and the carbon rod was placed at the cathode. Anodizing reaction at voltage of 30, 25, and 20 V were performed for 2 h.

### HHC-36 Coating Preparation

The prepared titanium dioxide nanotube specimens were shaken by ultrasonic in acetone, anhydrous ethanol, and deionized water for 15 min. After being taken out, the specimens were soaked in 75% alcohol in ultraviolet disinfection overnight and were placed on a clean bench for drying. Then 2.0 g PDLLA was precisely weighed and dissolved in 40 ml ethyl acetate. After ultrasonic vibration, 0.1 g HHC-36 antimicrobial peptide (AMP) was added into the solution, and 5% (AMP/PDLLA) suspension was obtained with ultrasonic vibration. The prepared TiO_2_ nanotube specimen was drip-loaded with liquid suspension until it was covered. The specimen was then allowed to stand for 15 min until ethyl acetate was completely volatilized; the procedure was performed thrice. The specimens were dried at room temperature for further study.

Then, 100 mg HHC-36 was dissolved in 0.4 ml methanol at 50°C for 30 s to form internal water phase, and 200 mg PLGA was dissolved in 2 ml dichloromethane which 0.2 ml surfactant span85 was added to form oil phase. The internal water phase was added into the oil phase, and the uniform milky solution was formed after ultrasonic treatment for 30 s. The solution was sealed to prevent volatilization. Then 1 ml hydrophilic surfactant Tween 20 was added into 120 ml 1% sodium alginate to form the external water phase. The milky white solution was injected into the external water phase with a syringe at a uniform speed. During the process, magnetic stirring was carried out at 1,200 rpm. After injection, stirring was carried out at 700 rpm for 4 h to make dichloromethane volatilize completely. After centrifugation at 10,000 rpm for 15 min and freeze-drying, the drug loaded nanoparticles were obtained. The suspension was obtained by resuspension of distilled water. Then the TiO_2_ specimens was dripped with the suspension, which were dried for 1 h at room temperature and repeated for three times. Then, all the specimens were dried overnight at room temperature. For quantitative determination of HHC-36, PDLLA-HHC-36 specimens (*n* = 5) and PLGA-HHC-36 specimens (*n* = 5) were vibrated by ultrasound in a mixed solution of 20 ml dichloromethane and 20 ml PBS. Then the absorbance of the samples was measured by spectrophotometer.

### Field Emission Scanning Electron Microscopy/Energy Dispersive X-Ray Analysis

Experiments were performed as previously indicated (Wang et al., 2014). Briefly, the processed specimens in each group were subjected to ultrasonic shaking in acetone, anhydrous ethanol, and deionized water in turn for 15 min; the specimens were then dried, observed using field emission scanning electron microscopy (FE-SEM, Hitachi JSM-4800, Japan), and the surface element was evaluated using energy dispersive x-ray analysis (EDAX).

### Antibiotic Release Test

HHC-36 sustained-release PDLLA and PLGA specimens were placed in 20 ml PBS at 37°C in a water bath. Then, 2 ml of the solution was taken out at different time points, and 2 ml PBS solution was replenished. Samples were taken once every hour on the first day for a total of six times; on the second to the 15th day, the samples were taken out at 11 AM every day. The samples were subjected to measurements using an ultraviolet spectral spectrophotometer, and the corresponding antimicrobial peptide content was calculated as per the regression equation. The data were sorted out, and the release curve *in vitro* was drawn.

### Cell Culture

Human primary dermal fibroblasts were obtained from ATCC (PCS-201-012). The cells were cultured in Dulbecco’s minimum Eagle’s medium (Hyclone) supplemented with 10% bovine calf serum (Gibco) and antibiotics (penicillin 100 U/mL and streptomycin 100 μg/ml; Sigma) in a humidified atmosphere with 5% CO_2_ and 95% air at 37°C. The cells from passages 2–5 were used.

### Cytotoxicity Test

Extracts of HHC-36-coated PDLLA/PLGA comprised the experimental group, while the extracts of PDLLA/PLGA formed the control group. Cells with no extract made up the blank control group. The extracts were added in cell suspensions for 12 and 24 h. Cell proliferation was measured using the CCK-8 method as per the manufacturer’s instruction (Beyotime biotechnology, Beijing, China).

### Immunofluorescence

For immunofluorescence analysis, the cells were treated as previously described and incubated with primary antibodies: anti-actin (#3700, Cell Signaling Technology). Secondary antibodies conjugated with fluorescent dyes (Jackson Immuno Research Laboratories) and 0.1 mg/ml DAPI were subsequently used for revealing. Images were collected using fluorescence microscopy (FV1000, Olympus, Japan) and analyzed with the Image Pro Plus software. For each image, 0.15 × 0.15-mm areas (*n* = 5) from the defect site were randomly selected, and the percentage of positive cells was calculated (for each group, *n* = 6).

### Bacterial Experiments

*Staphylococcus aureus* (ATCC25923) obtained from the microbiology laboratory of the Stomatology School, the Fourth Military Medical University was used as the indicator bacteria. Two independent colonies were selected for passage, crossed, and inoculated on blood AGAR plate, and cultured for 24 h in a 5% CO_2_ incubator at 37°C.

### Inhibition Zone Evaluation and Antibacterial Test

Two separate colonies of *Staphylococcus aureus* were cultured into the turbidimetric tube, and the concentration of 1 × 10^8^ CFU of *Staphylococcus aureus* was diluted sing the McBurnius turbidimetric method. The diluted *Staphylococcus aureus* solution was evenly spread on an MH AGAR plate using cotton swabs. Extracts of HHC-36-coated PDLLA/PLGA comprised the experimental group. Extracts of PDLLA/PLGA were included as controls. Cells with no extract were used as blank controls. Each group was placed on MH AGAR plates uniformly coated with *Staphylococcus aureus* bacteria liquid on a super-clean bench, and cultured in a 5% CO_2_ incubator at 37°C for 24 h. Thereafter, the size of the bacteriostatic circle was observed and measured. The specimens were cultured in a 5% CO_2_ incubator at 37°C for another 10 days, and the changes in the bacteriostatic circle were observed and recorded.

Single colony of *Staphylococcus aureus* was cultured in 80 ml BHI medium at 37°C for 24 h. The gradient concentrations of 1 × 10^5^, 10^4^, 10^3^ and 10^2^ CFU were obtained by Macintosh turbidimetry. The drug loaded specimens of each group were vibrated in 5 ml BHI culture medium for 30 min, and 1 ml of each group was mixed with gradient concentration bacterial solution. After bathing in 37°C water for 3 h, 100 μL of each bacterial solution was evenly inoculated on the blood agar plate and cultured in 5% CO_2_ incubator at 37°C for 24 h. Then the number of colonies was recorded.

### Animal Experiments

Male C57BL/6J mice were obtained from the Laboratory Animal Research Center of Fourth Military Medical University, Xi’an, China and used for all the *in vivo* experiments. The mice are kept under SPF conditions. The animal protocols were approved by the Institutional Animal Care and Use Committee of the University and fulfilled the National Institute of Health guidelines for the care and use of laboratory animals. All the experiments were performed as per the applicable guidelines and regulations.

Six mice were included in each of the following groups: the PDLLA-AMP group, PLGA-AMP group, and control group. Surgical instruments were sterilized using conventional high-temperature and high-pressure methods. After anesthesia administration, a dehairing agent was used to remove the bilateral femoral hair. Two femoral incisions were made to expose the bone surface. One implant on each side was implanted randomly in the experimental and the control group. Two weeks after the implantation, the animals were sacrificed; the soft tissue reaction around the implant was evaluated based on the evaluation criteria proposed by Holgers, K.M. (Table 1). The surrounding tissues, about 2 cm in length, were collected and fixed for 10 days in 10% neutral formalin solution. The samples were dehydrated and embedded; then, the paraffin sections were prepared. The sections were then stained with HE and analyzed using immunohistochemistry to observe the histomorphology.

**TABLE 1:** Peri-implant tissue response score.

Peri-implant tissue response	Score
Inapparent	0
Slight redness and swelling	1
Redness and swelling	2
Proliferation of granulation tissue	3
Infection	4

For immunohistochemistry, the sections were blocked with 1.5% goat serum (MilliporeSigma) and incubated with primary antibodies anti-IL-6 (ab208113, Abcam, Cambridge, United Kingdom). For the negative control sections, non-immune goat serum was substituted for the primary antibody. The sections were then incubated with peroxidase-conjugated secondary antibody (Jackson Immuno Research Laboratories, Inc.), visualized with 3,3-diaminobenzidine solution (Substrate Kit1, Vector Laboratories, Inc., Burlingame, CA, USA), and counter-stained with hematoxylin. Digitized images obtained from the stained sections were subjected to analyses with the Image Pro Plus software. For each obtained image, six areas were randomly selected (0.15 mm × 0.15 mm) from the defect area for determining the percentage of positive cells.

### Statistical Analyses

All the collected data were analyzed using SPSS 17.0 software (SPSS, USA). One-way ANOVA followed by a SNK post hoc test and paired *t* test was used to determine the level of significance; *p* value < 0.05 was considered to indicate statistical significance.

## Results

### Morphology and Surface Elemental Distribution of PDLLA/PLGA-HHC-36-Coated Titanium Nanotube

**FIGURE 1:**
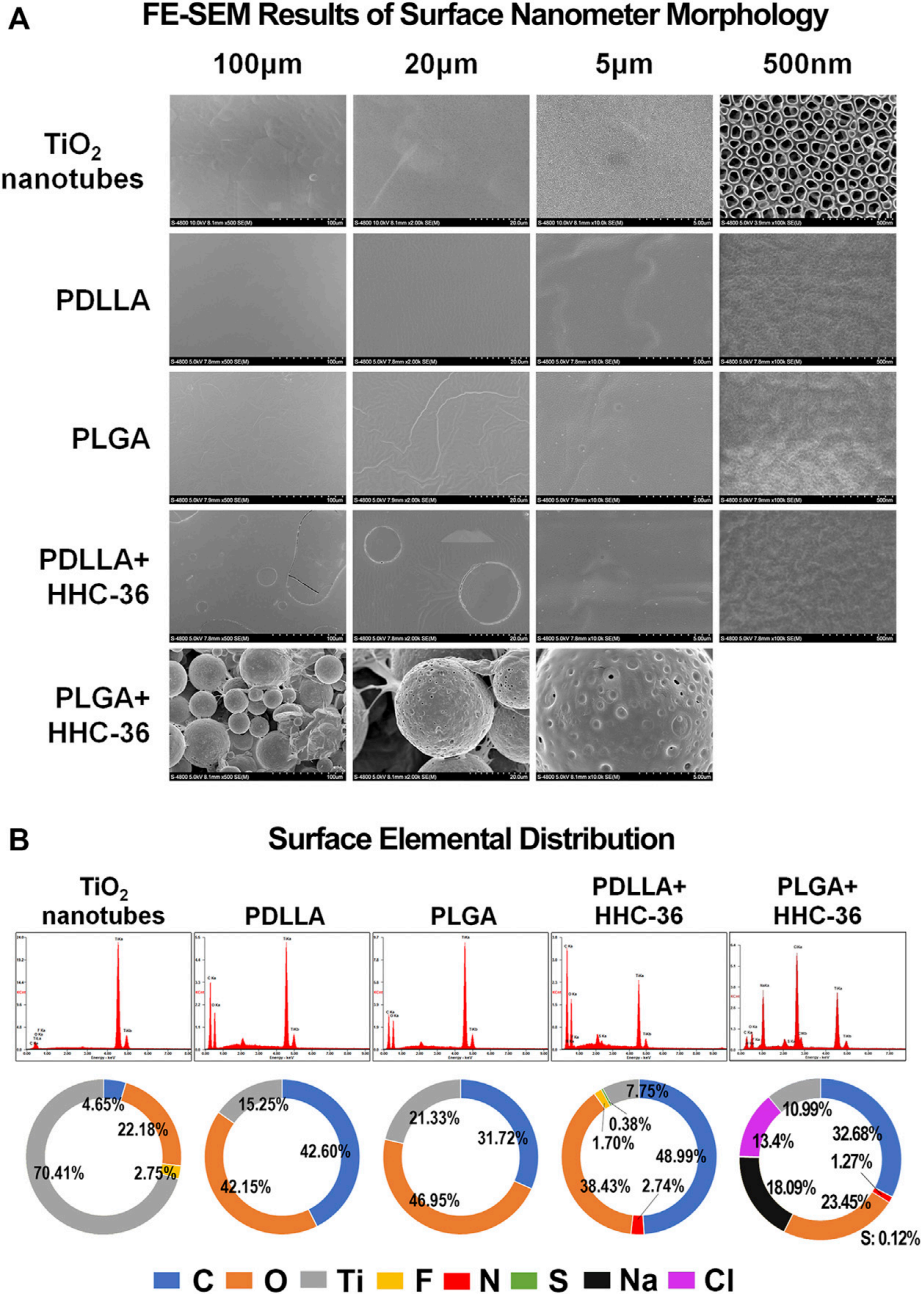
Morphology and surface elemental distribution of the PDLLA/PLGA-HHC-36 coated titanium nanotube. (**A**) Field emission scanning electron microscopy results of surface nanometer morphology. (**B**) Surface elemental distribution of un-coated titanium nanotube group (control group), PDLLA/PLGA group, and PDLLA/PLGA-HHC-36 group.

The characterization of the morphology of the prepared titanium nanotube samples before and after polymer coating have been shown in Figure 1A. Anodizing experiments were performed on round and columnar pure Ti specimens under 25 V. The SEM images of the top surface showed that the diameter of the tubes was 100 ± 20 nm, and the array was well arranged and well aligned. Images of the top surface of PDLLA/PLGA-HHC-36-coated samples showed a smooth and compact drug coating, with porous drug-loaded nanospheres on the titanium nanotube. Un-coated titanium nanotube substrates showed orderly arrangement of the nanotubes, and PDLLA/PLGA were coated on the specimen evenly and compactly. Further, 500 nm of PLGA-HHC-36 group could not be shown as spheroidal surface condition.

There was a large difference between the control and experimental groups in terms of surface elemental distribution (Figure 1B). In both, the PDLLA and PLGA groups, the content of carbon and oxygen was increased significantly, while the content of fluorine was reduced. In the PDLLA-HHC-36 group and PLGA-HHC-36 group, the percentage of carbon further increased.

### Biocompatibility of PDLLA/PLGA-HHC-36-Coated Titanium Nanotube

**FIGURE 2:**
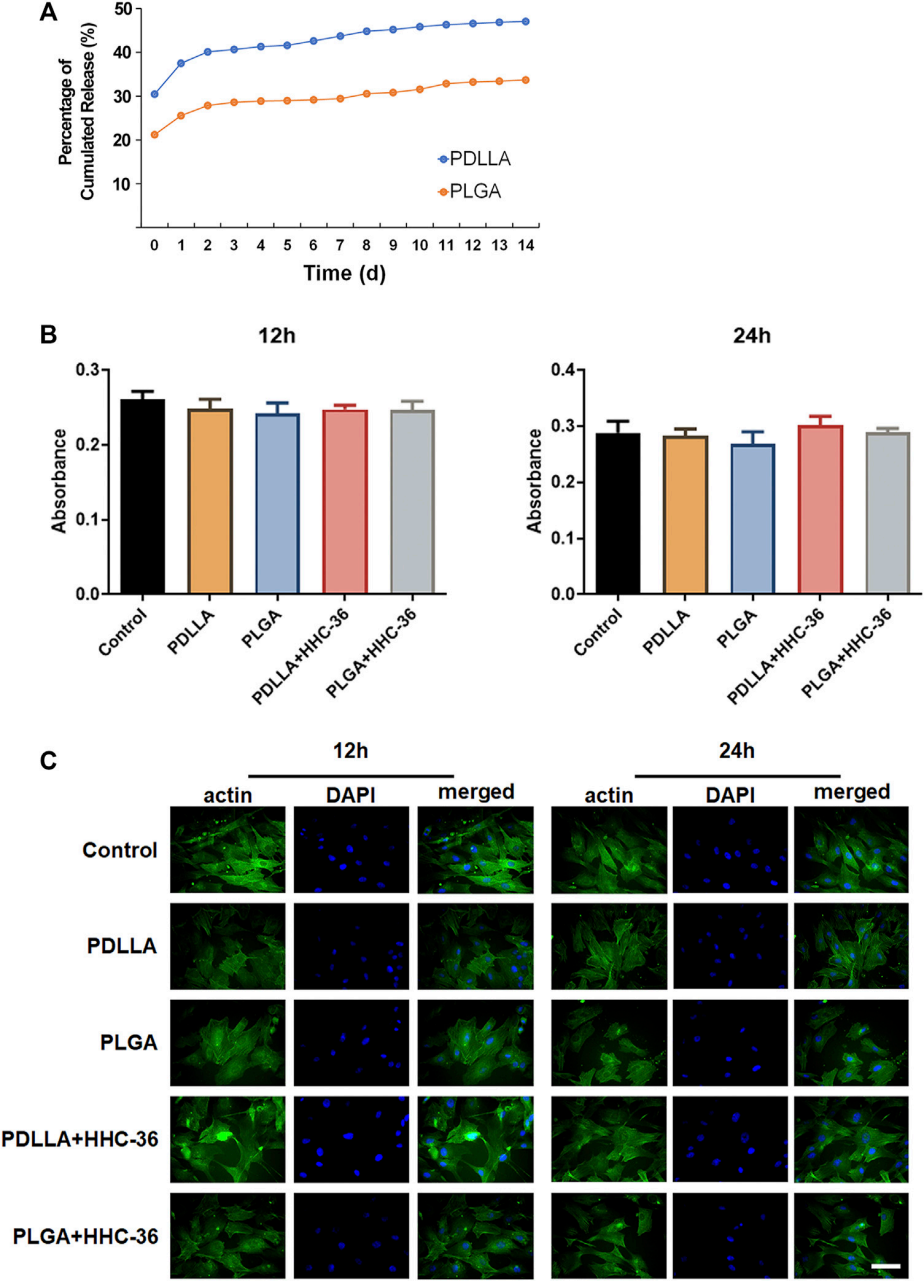
Biocompatibility of the PDLLA/PLGA-HHC-36 coated titanium nanotube. (**A**) Kinetics of *in vitro* release of HHC-36 with PDLLA/PLGA coatings. (**B**) Cell cytotoxicity examination using the CCK-8 test. Adult dermal fibroblasts cells intervened by eluents of each group were evaluated at 12 and 24 h. (**C**) Immunofluorescence results of actin expression at 12 and 24 h.

For biocompatibility evaluation, HHC-36 release curves *in vitro* were examined. The results showed that a burst release of HHC-36 was present in both PDLLA-HHC-36 coating specimens and PLGA-HHC-36 coating specimens in the first few hours (Figure 2A), with a release rate of 30% in the PDLLA group and 21% in the PLGA group on the first day. The release rates of the PDLLA and PLGA groups reached 47 and 33%, respectively, after 15 days. For quantitative determination of HHC-36, the average HHC-36 loading in PDLLA group was significantly higher than that in PLGA group (*p* < 0.05) (Supplementary Figure S2).

Cell cytotoxicity was examined using the CCK-8 test (Figure 2B). Results showed that after 12 and 24 h, there was no significant difference between the experimental group and the blank control group (*p* > 0.05). The toxicity grade (RGR = absorbance value of the experimental group/absorbance value of the control group) was grade 1 or grade 0, indicating no influence of the PDLLA/PLGA-HHC-36-coated titanium nanotube on cell proliferation (Supplementary Table S1).

Cytoskeleton staining of actin labeled by FITC-phalloidine was also performed to determine the biocompatibility of the specimens (Figure 2C). We found that HHC-36 antimicrobial peptide and its sustained-release agent could not alter cell migration and colonization, based on a comparison of the experimental and control groups. After culturing for 12 and 24 h, obvious tension fibers and actin microfilaments were observed on the surface cells of all the groups, indicating that the coating did not have a significant effect on the cell colonization on the surface of titanium dioxide nanotubes.

### Antibacterial Property of PDLLA/PLGA-HHC-36-Coated Titanium Nanotube *in vitro*

**FIGURE 3:**
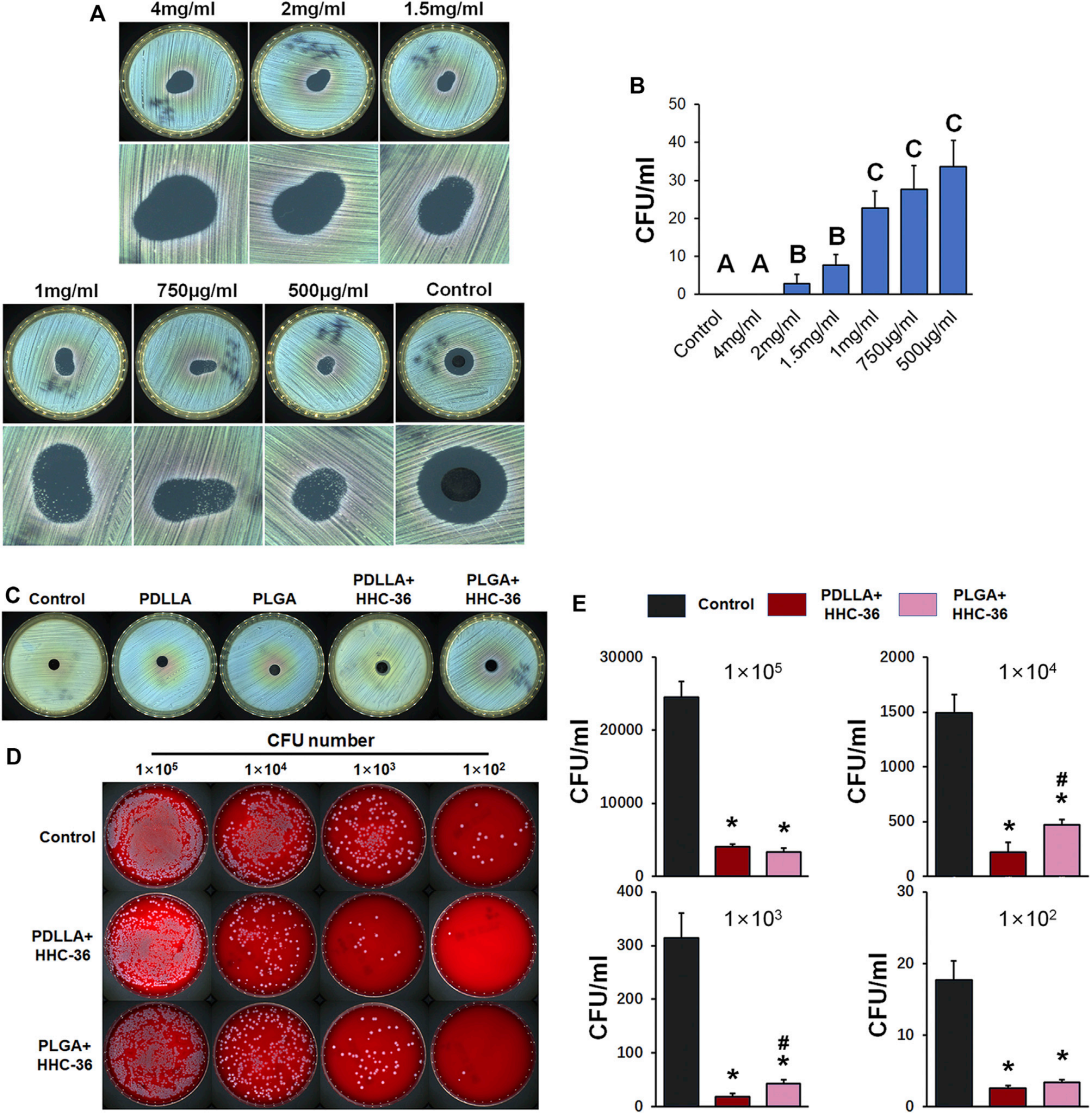
Antibacterial property of PDLLA/PLGA-HHC-36 coated titanium nanotube *in vitro*. (**A**) Inhibition zone test to determine the minimum inhibitory concentration of HHC-36. (**B**) *Staphylococcus aureus* CFU concentration with different doses of HHC-36 at 12 h. Groups labeled with different uppercase letters are significantly different (*p* < 0.05). (**C**) Inhibition zone test of un-coated titanium nanotube (control group), PDLLA/PLGA group, and PDLLA/PLGA-HHC-36 group. (**D, E**) Antibacterial property of eluent of PDLLA/PLGA-HHC-36 coated titanium nanotube. *p < 0.05 vs. Control counterparts. #*p* < 0.05 vs. PDLLA + HHC-36 counterparts.

We determined the minimum inhibitory concentration of HHC-36. Our results showed significant antibacterial effect of HHC-36 antibacterial peptide (Figures 3A,B). *Staphylococcus aureus* grows in the drug solution circle when the concentration of antimicrobial peptide is too low (0.5–1 mg/ml). However, when the concentration of the antimicrobial peptide reached the limit, negligible amount of *Staphylococcus aureus* grew in the drug solution circle (1.5–4 mg/ml). We determined that the minimum inhibitory concentration range of HHC-36 antimicrobial peptide was 1.5–2 mg/ml. This result was confirmed using the inhibition zone test (Figure 3C) of HHC-36 loaded PDLLA/PLGA specimens as per which, the inhibition zones of PDLLA/PLGA-HHC-36 coated titanium nanotubes were significant.

In addition, we evaluated the antibacterial property of the eluent of PDLLA/PLGA-HHC-36-coated titanium nanotubes (Figures 3D,E). Results showed that PDLLA/PLGA-HHC-36-loaded specimens exerted a significant inhibitory effect on the growth of *Staphylococcus aureus* at a concentration gradient of 1 × 10^5^, 1 × 10^4^, 1 × 10^3^, and 1 × 10^2^ CFU. This indicated that the sustained-release agent from HHC-36 antimicrobial peptide loaded on the surface of the sample retained its antimicrobial activity. Further, the PDLLA-HHC-36 group showed significantly lower CFUs at 1 × 10^3^, 1 × 10^4^, and 1 × 10^5^. For HHC-36 in PDLLA group was all dispersed in eluent, and antimicrobial peptide in PLGA group needed time to release. Moreover, this finding indicates that the antibacterial effect of HHC-36 is dose-dependent.

### Antibacterial Property of PDLLA/PLGA-HHC-36 Coated Titanium Nanotube *in vivo*

**FIGURE 4:**
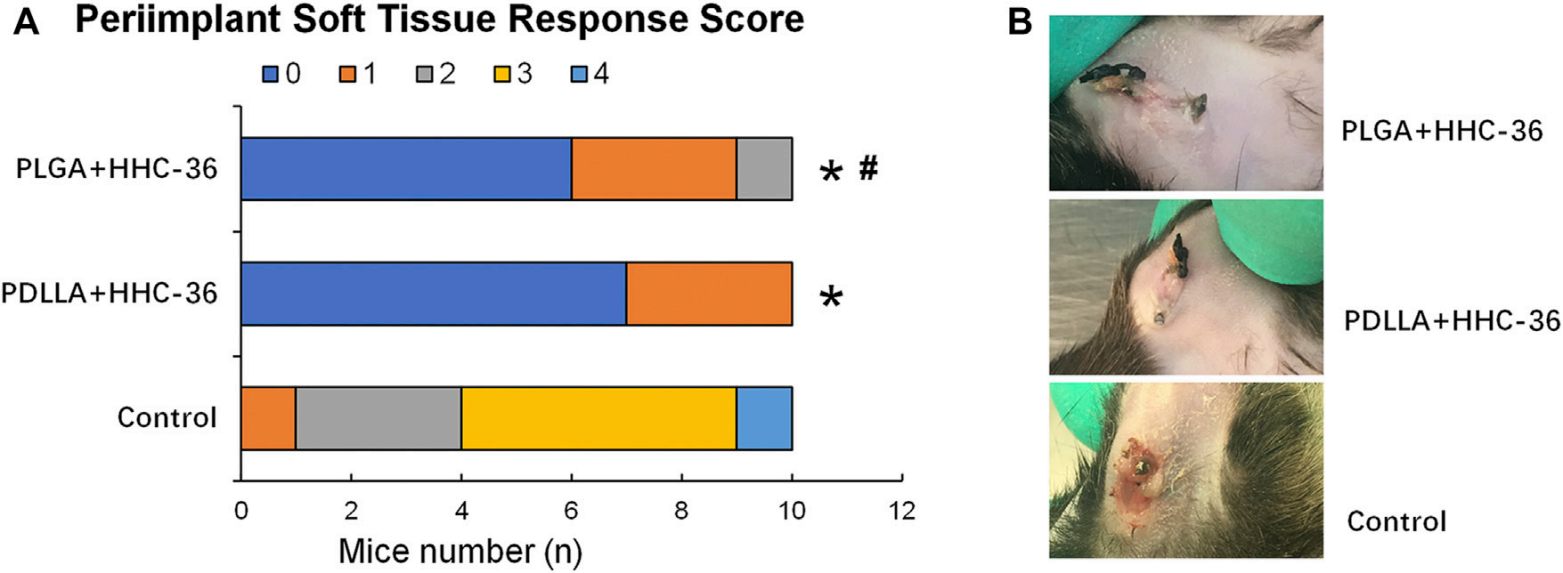
Results of percutaneous implant mice model 2 weeks postoperatively. (**A**) Peri-implant soft tissue response score. (**B**) Comparison of tissue infection around the implants in different groups of mice.

A percutaneous implant mouse model was established to evaluate the antibacterial property of PDLLA/PLGA-HHC-36-coated titanium nanotubes *in vivo*. The soft tissue reaction around the implant (Figures 4A,B) showed that the peri-implant soft tissue response score in both, the PDLLA and PLGA groups was significantly higher than in the control group (*p* < 0.05). Moreover, the score of the PDLLA group was significantly higher than that of the PLGA group, indicating that the antibacterial property of PDLLA-HHC-36-coated titanium nanotubes was potentially superior.

**FIGURE 5:**
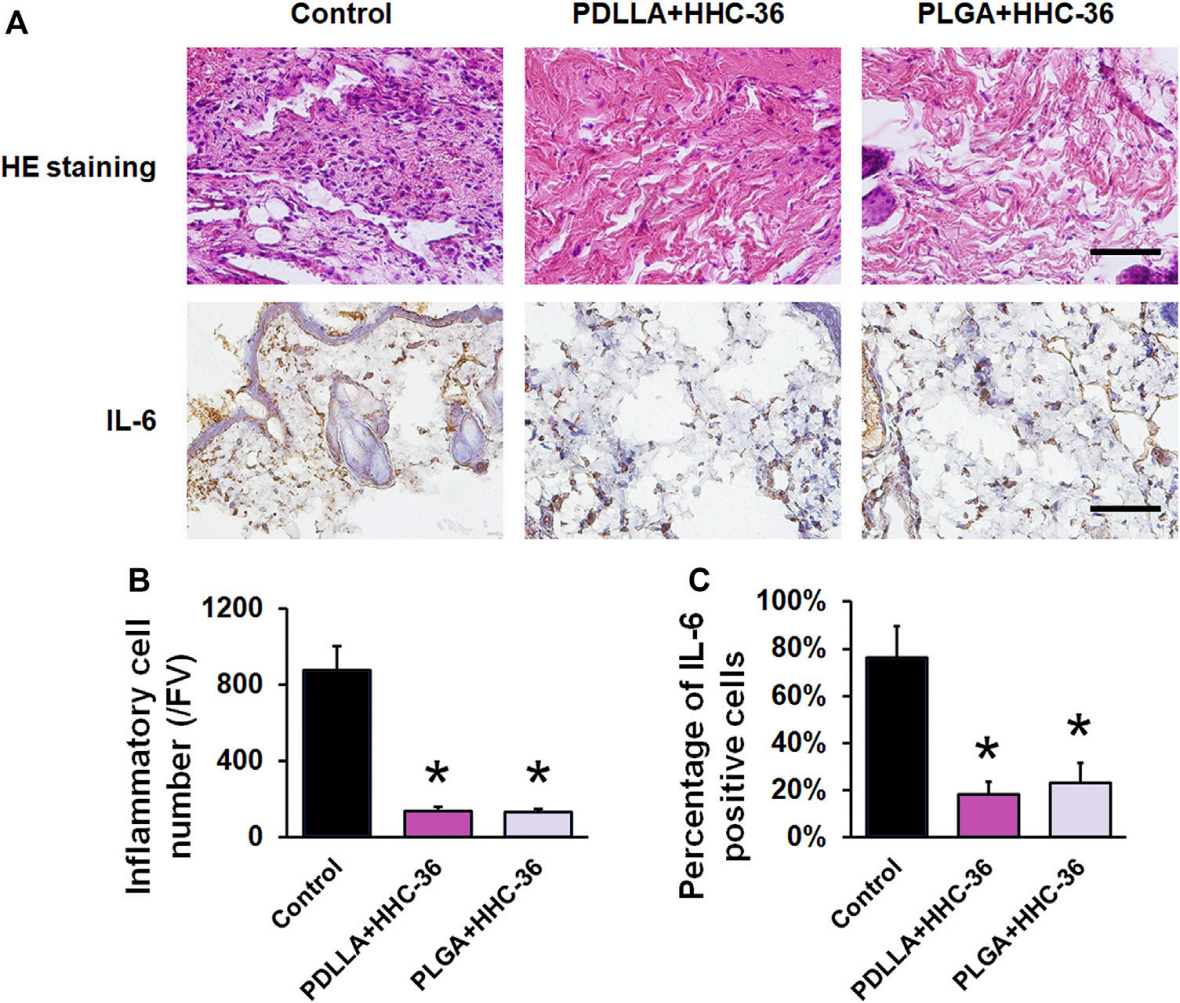
Evaluation of the infection indicators *in vivo*. (**A**) H&E staining and IL-6 immunohistochemical staining. Bar = 200 μm. (**B**) Inflammatory cell number/FV of H&E staining. (**C**) Semiquantitative results of IL-6 positive cells. *p < 0.05 vs. the controls.

The results of H&E staining and IL-6 immunohistochemical staining (Figure 5A) also confirmed the antibacterial effects in both, the PDLLA and PLGA groups. H&E staining showed that the inflammatory cells in the PDLLA and PLGA groups were significantly decreased as compared to that in the control group (*p* < 0.05) (Figure 5B). Moreover, the results of IL-6 immunohistochemical staining showed that the percentage of IL-6 positive cells was significantly lower than that in the control group (*p* < 0.05) (Figure 5C).

## Discussion

The preparation process of titanium dioxide nanotubes is simple and suitable for the formation of coating on the surface of complex objects, such as implants (Mendonça et al., 2008). The surface nanotube structure can improve cell adhesion and proliferation to enable better integration with the skin and promote transdermal closure (Puckett et al., 2010; Farrell et al., 2014b). The nanotube structure can be used to carry drugs, and the nanotube structure itself possesses certain antibacterial properties (Tan et al., 2015). However, the skin environment in which the percutaneous implants are located is prone to bacterial adhesion, and bacterial invasion commonly occurs after percutaneous implantation even after strict disinfection and infection control (Kaiser 1986). Percutaneous infections are mainly caused by *Staphylococcus aureus* and coagulase-negative *staphylococcus* (von Eiff et al., 2005; Zimmerli and Ochsner 2003). In our study, the percutaneous implant was surrounded by a bacterial biofilm during the healing process. Therefore, it was not enough to change the surface morphology of the percutaneous implant to meet the antibacterial standard. Research has shown that the initial bacterial adhesion is a key event in the pathogenesis of peri-implant infection (Busscher et al., 1995); thus, it is important to have knowledge regarding the method for inhibiting initial bacterial adhesion after percutaneous implant implantation.

Many researchers have attempted to construct antimicrobial coatings on implant surfaces (Stigter et al., 2004; Alt et al., 2006). Antimicrobial peptides are usually amphiphilic cationic peptides with a broad antibacterial spectrum. Their antibacterial mechanism mainly involves the disruption of the bacterial cell membrane and inhibition of the synthesis and expression of biological macromolecules (Brogden 2005); this makes it difficult to produce drug-resistant strains (Domingues et al., 2012) and cause low cytotoxicity in mammals (Ballweber et al., 2002), making them an ideal antimicrobial agent. As per a recent large quantitative structure-activity relationship study, HHC-36, a cationic antimicrobial peptide had a strong broad-spectrum antibacterial effect (Cherkasov et al., 2009). The antibacterial activity test showed that HHC-36 could effectively kill *Staphylococcus aureus* and Gram-positive bacteria, with the killing rate of the surrounding bacteria reaching 99.9% after loading on the surface of titanium dioxide nanotubes; further, it could effectively reduce the total number of bacteria attached to the surface after 4 h (Ma et al., 2012). Studies have also shown that HHC-36 exerts a strong bactericidal effect on *Pseudomonas aeruginosa* (Kazemzadeh-Narbat et al., 2013). To our knowledge, the present study is the first to demonstrate that HHC-36 coated titanium nanotubes could improve the antibacterial potential of percutaneous implants.

Although the implant itself possesses a certain drug-loading ability, it generally has no controlled release ability; this makes it difficult to maintain the minimum inhibitory concentration in the high-risk postoperative infection period for a certain time. Therefore, several trials have attempted to use certain other methods for controlling drug release. High molecular polymers, such as PDLLA and PLGA, have good biocompatibility and can generally be degraded into non-toxic irritant substances for the body. These also represent the most commonly used class of sustained-release agents at present (Shive and Anderson, 1997). PDLLA, as a sustained-release material of implant coating, offers not only good biocompatibility, but also exhibits favorable mechanical property and anti-thrombus potential (Gollwitzer et al., 2003). PLGA coating also achieved sustained release for up to 26 days and possesses good antibacterial properties (Kumeria et al., 2015). In the present study, PDLLA and PLGA coating showed sustained release of HHC-36 for up to 14 days and effectively prevented infections in a mouse model. Though the adhesivity of the coatings were not specifically examined in the present study, the specimens implanted into mice for 2 weeks showed obvious antibacterial properties in the animal experiments, indicating that the adhesivity of PDLLA and PLGA is stable and sufficient.

The present study has certain limitations. The antibacterial properties of different bacteria in the complex environment around the skin and *in vivo* animal experiments require to be studied in further experiments to evaluate the application of PDLLA/PLGA-HHC-36-coated titanium nanotube in percutaneous implantation.

## Conclusion

Nanotube structure on the surface of the titanium dioxide nanotubes facilitates the integration of the percutaneous implant with the skin accessory. The present study demonstrated that with antibacterial peptide HHC-36 sustained-release coating, titanium dioxide nanotubes maintain effective drug release for 15 days *in vitro*, and show significant antibacterial activity. Besides, PDLLA-HHC-36 and PLGA-HHC-36 coating was demonstrated to be biocompatible and antibacterial *in vivo*. The results of the present study indicated a novel and efficient strategy in preventing bacterial infection of percutaneous implants.

## Data Availability

The raw data supporting the conclusion of this article will be made available by the authors, without undue reservation.
