# Supplementary material for: Antibacterial Peptide HHC-36 Sustained-Release Coating Promotes Antibacterial Property of Percutaneous Implant
Source: Front Bioeng Biotechnol. 2021 Sep 27;9:735889. doi: 10.3389/fbioe.2021.735889 (PMC8503604; doi:10.3389/fbioe.2021.735889)
Supplement: Supplementary file 1 [file DataSheet1.DOCX]

**Antibacterial peptide HHC-36 sustained-release coating promotes antibacterial property of percutaneous implant**

Qiang Miao^1,2,#^, Jin-Long Sun^1,#^, Huang Fei^1^, Jing Wang^3^, Pei Wang^4^, Ya-Fei Zheng^2^, Feng Wang^1,^*, Chu-Fan Ma^2,3,^*

^1^Department of Stomatology, Sixth Medical Center of PLA General Hospital, Beijing 100048, China.

^2^State Key Laboratory of Military Stomatology & National Clinical Research Center for Oral Diseases & Shaanxi Key Laboratory of Stomatology, School of Stomatology, The Fourth Military Medical University, 145 changle xi road, Xi’an, Shaanxi, 710032, China.

^3^Air Force Medical Center, The Fourth Military Medical University, 30 FuCheng Road, Beijing, 100089, China.

^4^Department of Bone and Joint Surgery, Wendeng Orthopaedic Hospital of Shandong Province, Wendeng, Shandong, 264400, China.

^#^These authors contribute equal to this work.

*Corresponding authors: Chu-Fan Ma, E-mail: [machufan_fmmu@163.com](mailto:machufan_fmmu@163.com); Feng Wang, E-mail: [wolfwang2003@aliyun.com](mailto:wolfwang2003@aliyun.com).


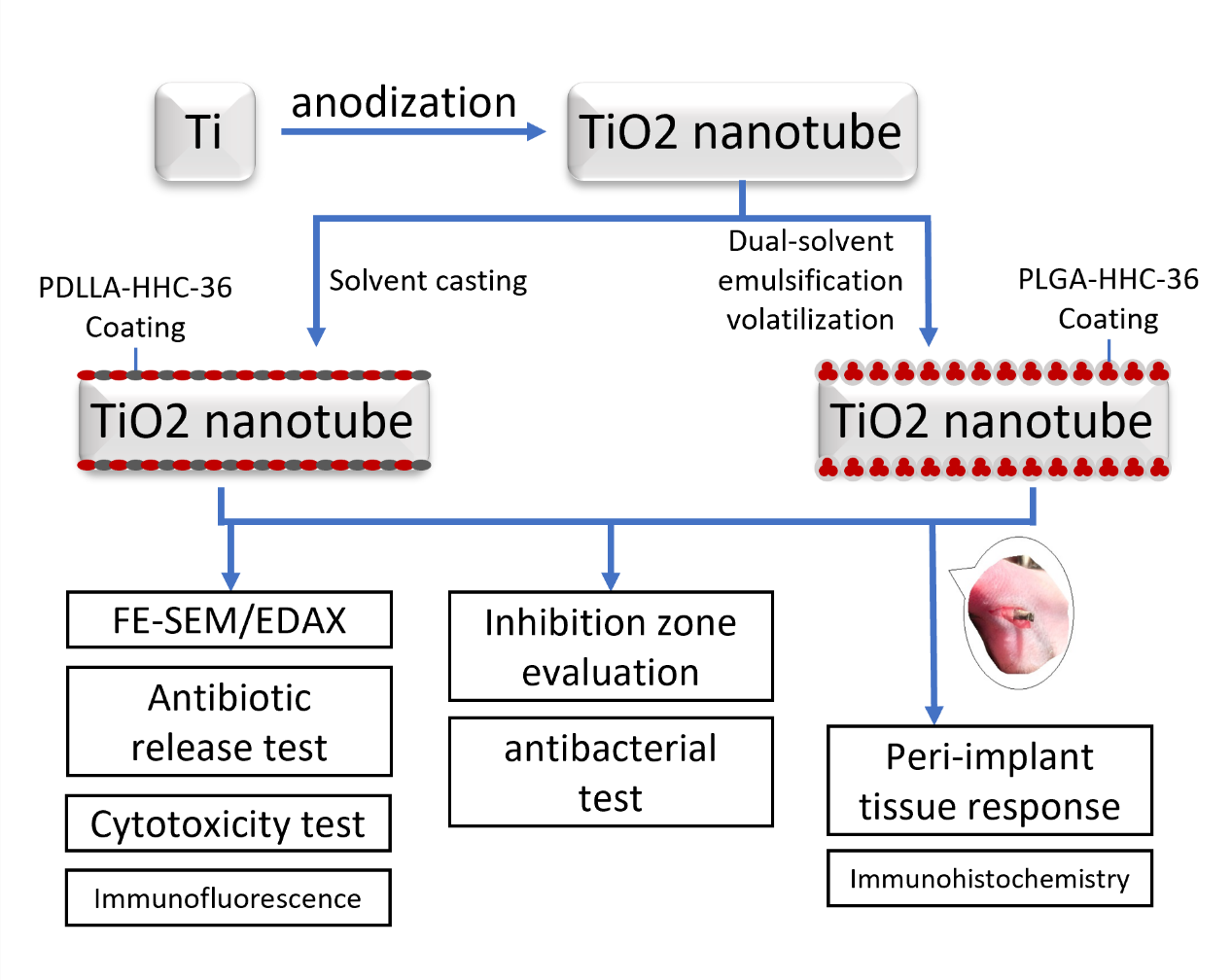


**Supplementary Figure 1.** Flow chart depicting the sequence of experiments conducted in the present study.

**
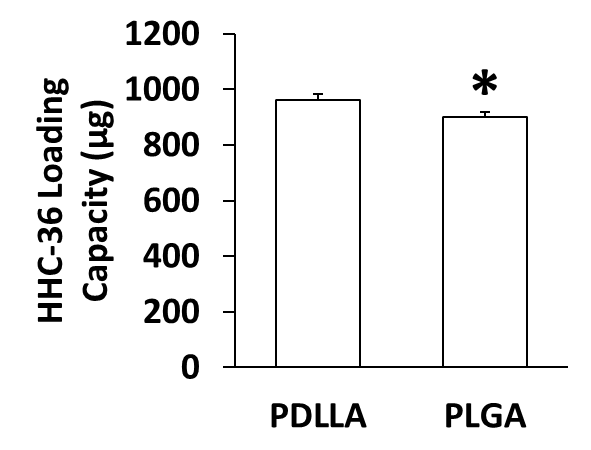
**

**Supplementary Figure 2.** Quantitative determination of HHC-36 loaded on PDLLA and PLGA coated titanium dioxide nanotubes. *P<0.05

**Supplementary Table 1.** Result of CCK-8 test

| Group | Absorbance | | RGR（%） | | Toxicity Level | |
| --- | --- | --- | --- | --- | --- | --- |
|  | 12 h | 24 h | 12 h | 24 h | 12 h | 24 h |
| Control | 0.264±0005 | 0.286±0019 | 100 | 100 | 0 | 0 |
| PDLLA | 0.251±0.008 | 0.283±0.013 | 95.1 | 98.95 | 1 | 1 |
| PLGA | 0.242±0.008 | 0.283±0.038 | 91.7 | 98.95 | 1 | 1 |
| PDLLA+HHC-36 | 0.248±0.011 | 0.302±0.018 | 93.9 | 105.6 | 1 | 0 |
| PLGA+HHC-36 | 0.247±0.016 | 0.286±0.002 | 93.6 | 100 | 1 | 0 |

h: hours. RGR: relative growth rate. Toxicity Level was rated according to the National Formulary The United States Pharmacopeia.
